# Interesting Electrophysiological Findings in a Patient With Coincidental Right Ventricular Outflow Tract and Atrioventricular Nodal Reentrant Tachycardia

**Published:** 2004-04-01

**Authors:** Bulent Ozin, Bahar Pirat, Haldun Muderrisoglu

**Affiliations:** Baskent University Faculty of Medicine, Department of Cardiology, Ankara, Turkey

**Keywords:** Non-sustained ventricular tachycardia, supraventricular tachycardia, double tachycardia

## Abstract

Tachycardia induced tachycardias are not common in clinical practice, and it is believed that most cases of double tachycardia are coincidental. The existence of two different tachycardias in the same patient almost always poses problems in the electrophysiology laboratory. However, in rare instances, the emergence of a second tachycardia can actually provide invaluable information about the first one. In this report, we describe a 30-year-old woman who presented with palpitations. Electrophysiological study revealed that atrial programmed stimulation at baseline induced right ventricular outflow tract (RVOT) tachycardia and supraventricular tachycardia. The study also showed that each of the tachycardias was able to induce the other. A short run of RVOT tachycardia during supraventricular tachycardia was able to entrain the latter. This finding provided important information about the nature of the supraventricular tachycardia, which proved to be atrioventricular nodal reentrant tachycardia. Both of these tachycardias were successfully ablated, and the patient’s palpitations disappeared.

## Introduction

Simultaneous occurrence of supraventricular and ventricular tachycardia in the same patient is very rare. Concomitance of both these forms is usually coincidental, except in cases of digitalis toxicity and catecholaminergic tachycardias [1,2]. We report a case of double tachycardia in which right ventricular outflow tract (RVOT) tachycardia and atrioventricular nodal reentrant tachycardia (AVNRT) were capable of initiating each other.

## Case Report

A 30-year-old woman was referred to our hospital for evaluation of non-sustained ventricular tachycardia. She complained of palpitations lasting 20 to 30 minutes, and said she had had this problem for 2 years. Episodes of non-sustained ventricular tachycardia were documented during an exercise stress test. She was treated with verapamil and metoprolol with no success. Her physical examination was normal and an echocardiogram revealed no structural abnormalities. Electrocardiography showed frequent monomorphic ventricular premature beats of left bundle-branch block and inferior axis configuration.

An electrophysiological study was performed with the patient in postabsorptive, non-sedated state. An EP Tracer electrophysiology system (Maastricht, The Netherlands) was used. During the study, sinus rhythm was very frequently interrupted by spontaneous repetitive monomorphic non-sustained ventricular tachycardias (NSVT) with left bundle-branch block and inferior axis configuration. Mapping studies performed using an ablation catheter (Blazer II, Boston Scientific, USA) revealed that the ectopic activity originated from the RVOT. However, attempts to precisely map these premature depolarizations were frequently interrupted by a supraventricular tachycardia of cycle length 280 ms, which was started by the NSVT. The supraventricular tachycardia had a ventriculoatrial interval of 45 ms, which suggested either AVNRT or atrial tachycardia as the pathophysiologic mechanism.

**Figure 1:**
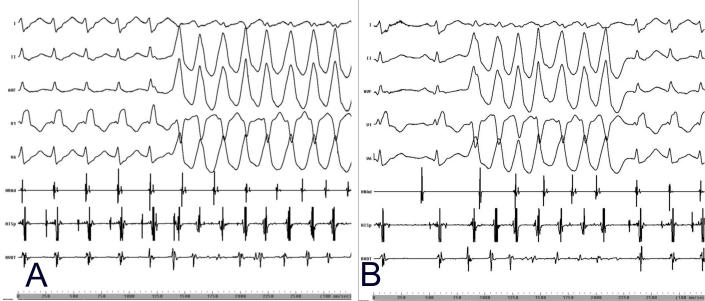
Initiation of the two tachycardias. **A.** Initiation of non - sustained ventricular tachycardia by the supraventricular tachycardia. **B.** Initiation of supraventricular tachycardia by the non-sustained ventricular tachycardia. Surface leads I, II, aVF, V1, V6 and intracardiac recordings from the high right atrium (HRAd), His bundle (HISp) and right ventricular outflow tract (RVOT) are shown.

**Figure 2:**
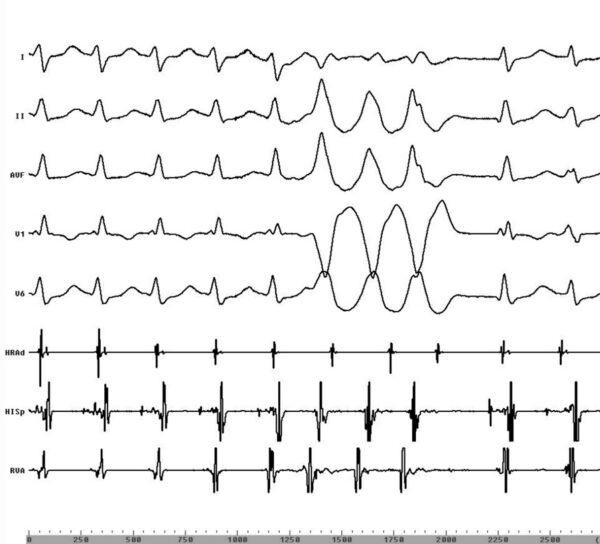
Entrainment of the supraventricular tachycardia by a short run of non - sustained tachycardia. Surface leads I, II, aVF, V1, V6 and intracardiac recordings from the high right atrium (HRAd), His bundle (HISp) and right ventricular apex (RVA) are shown.

Due to the frequent ventricular premature beats and runs of NSVT, it was not possible to further analyze and identify the exact nature of this supraventricular tachycardia. In order to continue mapping for the ventricular tachycardia, it was necessary to terminate the supraventricular tachycardia by overdrive pacing each time. There were occasions when the supraventricular tachycardia initiated the NSVT and vice versa (Figure 1 and B). An episode of NSVT during supraventricular tachycardia was observed to entrain the latter with a pattern compatible with AVNRT (Figure 2) [3].

The ventricular tachycardia was eliminated after 14 radiofrequency applications (EPT, USA) were delivered to the RVOT. Ventricular programmed stimulation after the ablation revealed no inducible ventricular tachycardias and demonstrated that the ventriculoatrial conduction was decremental. Atrial programmed stimulation showed dual atrioventricular nodal physiology and inducible AVNRT. A single radiofrequency application to the right posterior septum eliminated the dual nodal physiology and AVNRT. At a follow-up check 1 month after the procedure, the patient was free of palpitations.

## Discussion

Tachycardia induced tachycardia is a rare phenomenon that is only discussed in a few case reports in the literature. Wagshal and colleagues described one patient with idiopathic left ventricular tachycardia and AVNRT [4]. In that case, each of the tachycardias transformed into the other spontaneously. Washizuka and co-workers reported another patient who had RVOT tachycardia and atrioventricular reciprocating tachycardia [5]. Similar to what we observed in our case, the ventricular tachycardia in this patient was able to entrain the supraventricular tachycardia.

Cooklin and McComb recently reported a case in which AVNRT was induced by RVOT tachycardia during infusion of isoprenaline in an electrophysiological study [6]. Our patient also exhibited both AVNRT and RVOT tachycardia; however, in contrast to the case reported by Cooklin and McComb, both these tachycardias were capable of inducing each other. In addition, there are a few other discrepancies between that case and ours. First, our patient exhibited excellent ventriculoatrial conduction, which could easily explain the mechanism of induction of AVNRT during RVOT tachycardia. Second, neither isoprenaline nor any other beta-mimetic agents were used during our electrophysiological study, and induction of RVOT tachycardia by AVNRT took place at the basal state. It is well-known that triggered activity is the mechanism behind RVOT tachycardia, and rapid atrial pacing in a patient with good atrioventricular conduction might induce this form of tachycardia. In our case, we believe that RVOT tachycardia was induced by the rapid atrial depolarizations conducted to the ventricle during AVNRT.

The consecutive occurrence of two different tachycardias during electrophysiological testing always presents a major challenge for the electrophysiologist. During our electrophysiological study, we aimed to ablate the RVOT tachycardia first since it was responsible for the patient’s main symptoms. However, our attempts to locate the earliest ventricular activating sites during RVOT tachycardia were frequently interrupted by intervening AVNRT. To address this, we changed our strategy and decided to ablate the supraventricular tachycardia first, but the frequent premature beats and ventricular tachycardias arising from the RVOT prevented analysis of the mechanism of the supraventricular tachycardia. Thus, we had to change our plan again, and finally ablated the RVOT tachycardia after 14 radiofrequency applications.

Apart from the problems mentioned above, we observed that double tachycardias actually helped establish the diagnosis during electrophysiological study. In our case, a three-beat run of RVOT tachycardia during the supraventricular tachycardia entrained the latter with an ‡atrial-ventricular’ pattern, and helped us diagnose AVNRT (Figure 2).

In conclusion, this case is of particular interest because of the unusual finding of double tachycardia, with each form capable of inducing the other. The case is also noteworthy in that it highlights the advantages and disadvantages of dealing with two different tachycardias in the electrophysiology laboratory.
